# A Retrospective Study of thrombolysis with 0.6 mg/kg Recombinant Tissue Plasminogen Activator (rt-PA) in Mild Stroke

**DOI:** 10.1038/srep31344

**Published:** 2016-08-11

**Authors:** Jie Yang, Fei Yu, Hong Liu, Hedi An, Ran Xiong, Dongya Huang

**Affiliations:** 1 Department of Neurology, East Hospital, Tongji University School of Medicine, Shanghai 200120, China

## Abstract

We sought to assess the safety, effectiveness and cost of 0.6 mg/kg rt-PA treatment for patients with acute mild stroke and to compare that with 0.9 mg/kg. We retrospectively analyzed consecutive acute ischemic stroke patients who had a NIHSS score ≤5 at admission and who were treated with rt-PA within 4.5 hours of symptom onset. The demographic data, clinical outcomes and hospitalization cost were analyzed. A total of 108 patients were included. Forty six patients (42.6%) received a 0.6 mg/kg dosage of rt-PA. The baseline characteristics of the two groups were well matched (*p* > 0.05). Regarding the safety and effectiveness, the 0.6 mg/kg dosage group had a comparable proportion of symptomatic intracranial hemorrhage (sICH) (0.6 mg/kg, 4.3% vs 0.9 mg/kg, 4.8%; *p* > 0.05), early neurological deterioration (END) (19.6% vs 17.7%; *p* > 0.05), in-hospital mortality (4.3% vs 1.6%; *p* > 0.05), and a similar rate of favorable functional outcome (mRS score 0–1) at 3 months (73.9% vs 71.0%; *p* > 0.05) to those who received the standard dosage. However, the hospital cost was markedly lower in the 0.6 mg/kg group (0.6  mg/kg, 3,401.7 USD vs 0.9  mg/kg, 4,157.4 USD; *p* < 0.01). Our study suggest that 0.6 mg/kg rt-PA shared similar effectiveness and safety profile compared with that of 0.9 mg/kg in treating mild stroke, but cost less.

Patients with mild stroke deficits have previously been excluded from rt-PA administration, and it was not until recently that the newly released recommendations and statements have considered the necessity of rt-PA for this subgroup, especially for those with disabling symptoms; however, the opinions to treat patients with judged non-disabling stroke symptoms remain undetermined^1^,^2^.

It has been reported that mild or rapidly improving symptoms are the major reasons for withholding rt-PA from patients within the standard thrombolytic window^3^, as there are few randomized clinical trials reporting satisfactory risk-to-benefit ratios of rt-PA in this population. The published evidence has shown mixed results regarding these clinical outcomes, with some studies suggesting better functional outcomes^4^,^5^,^6^,^7^,^8^,^9^, while others demonstrating no further improvement^10^,^11^,^12^. However, although been constantly excluded from the treatment, the fact is about one-third of these patients not treated with rt-PA are left with substantial rates of disability after discharge^13^.

The main concern of administering a rt-PA treatment for mild stroke is the unnecessary risk of sICH. A few studies have shown that patients who received this treatment had a better clinical outcome accompanied by a higher, but not significant, risk of sICH, than patients who had a similar NIHSS score but without the same treatment. To maximize the risk-benefit profile, we must minimize the possible risk. Perhaps a lower dosage of rt-PA could provide comparable effectiveness to a full dose but reduce the sICH incidence and decrease the cost for these mild stroke patients. In October 2005, a similar approach was implemented in Japan, where the officially approved dosage of rt-PA was set at 0.6 mg/kg for eligible patients who have experienced an acute ischemic stroke^14^. Additionally, the safety profile of a lower dosage of rt-PA has been confirmed in the latest randomized, open, blinded endpoint clinical trial (ENCHANTED)^15^. Moreover, although the 0.6 mg/kg regimen has not yet been approved in Taiwan and mainland China, reports from both areas have shown that almost 50% of all acute ischemic patients treated with rt-PA have received a dose of <0.9  mg/kg^16^,^17^.

Thereby, the aim of this study was to compare the effectiveness, safety and in-hospital cost between the two dosages of rt-PA in patients who presented with mild stroke, defined as a NIHSS score ≤5^5^,^7^.

## Methods

### Patients

We conducted a single-center, retrospective analysis of all consecutive mild stroke patients who received rt-PA within 4.5 hours of symptom onset and whose clinical data were prospectively recorded in our stroke registry between January 2013 and January 2016. As commonly used in most previous studies, mild stroke was defined as a NIHSS score ≤5 at admission. The study protocol was approved by our institution’s ethics committee, and patient consent for the research was not required as it was a retrospective study.

### Clinical evaluation

The baseline demographic data, blood pressure (BP), serum glucose, low-density lipoprotein cholesterol (LDL-c), stroke subtype defined according to the Trial of Org 10172 in Acute Stroke Treatment (TOAST) categories^18^, NIHSS score at admission, latency between symptom onset and initiation of rt-PA, prognosis within the first 24 hours of rt-PA administration, presence of sICH, in-hospital cost, and mRS at 3 months were all collected. Brain CT scans were performed at admission and 24 hours after the administration of rt-PA as a part of the routine care or at any time when there were signs indicating altered condition, such as vomiting or a BP fluctuation. The patients with mild stroke were given 0.6 mg/kg rt-PA within 4.5 hours of onset (a 10% bolus, followed by a continuous infusion of the remainder over 1 hour) if they could not afford the cost of the standard dose (0.9 mg/kg), if they were considered to be at high sICH risk with the standard dose, or if they were estimated to have a high probability of achieving a full recovery spontaneously. The other patients received a standard dose of rt-PA (0.9 mg/kg body weight, up to a maximum amount of 90 mg).

### Clinical outcomes

The clinical outcomes included: 1. END, defined as an increase in the NIHSS score to ≥4 within the first 24 hours of rt-PA^19^; 2. Incidence of sICH, defined as the presence of any ICH confirmed by CT scan accompanied by neurologic worsening of the NIHSS score to ≥4 points^20^; 3. Favorable functional outcome, defined as an mRS of 0 or 1 at 3 months, which was also used as the primary outcome; 4. Cost of in-hospital treatment (from administration of rt-PA in the emergency room to discharge from hospital); and 5. In-hospital mortality.

### Statistical analysis

The quantitative variables were presented as a percentage (%), and the continuous data as 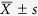 or the median and interquartile range. For categorical variables, statistically significant intergroup differences were assessed by a χ^2^ test; for continuous variables, the Mann-Whitney *U* test or Student’s *t* test was used. Multivariate logistic regression analysis was used to enhance the statistical power of the study and to test the independent effect of different dosages on the functional outcome at 3 months. Dose and other variables with *p* < 0.10 in the univariate analysis were entered into multivariate analysis. All *p* values were 2-sided, and statistical significance was defined as *p* < 0.05. All statistical analyses were performed with the SPSS19.0 statistical package.

## Results

### Demographic and baseline characteristics

A total of 116 consecutive mild stroke patients who received rt-PA were studied (none had received endovascular therapy). Of these, 8 patients were excluded as they had either a pre-morbid mRS score (n = 3) or were lost to follow up (n = 5). Of the remaining 108 mild stroke patients, 46 received 0.6 mg/kg rt-PA, while the other 62 patients received the standard, 0.9 mg/kg dose. The baseline characteristics of the two rt-PA dosage groups did not differ from each other, except that the subjects who received the 0.9 mg/kg dose had a lower (but not significantly lower) NIHSS score and a shorter latency between onset and rt-PA administration than those who received the 0.6 mg/kg dose (Table 1).

### Clinical course after thrombolysis

There was no significant difference in the proportion of patients who experienced END between the two groups (0.6 mg/kg, 19.6% vs 0.9 mg/kg, 17.7%; *p* > 0.05) (Table 2). Additionally, there was a non-significantly lower incidence of sICH in the 0.6 mg/kg dose group (0.6  mg/kg, 4.3% vs 0.9  mg/kg, 4.8%; *p* > 0.05) (Table 2). However, the two sICH patients in the 0.6 mg/kg dose group died days after the hemorrhage, while only one of the three sICH patients in the 0.9 mg/kg dose group suffered a fatal sICH. Additionally, the mortality in the 0.6 mg/kg dose group was higher than that in the 0.9 mg/kg dose group (4.3% vs 1.6%, respectively; *p* > 0.05). Furthermore, among the 3 systemic hemorrhages in the standard dosage group (not presented in the table), all were mild gum bleeding during thrombolysis (1case ceased the thrombolytic treatment with approximately 5 mg rt-PA left), while no patient developed this complication in the 0.6 mg/kg dose group.

### Functional outcome

**Figure 1:**
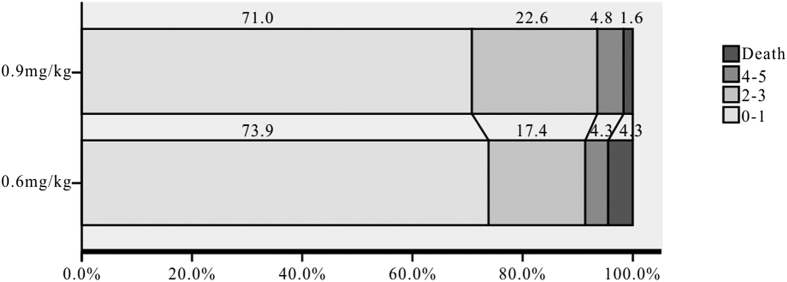
Distribution of mRS at 3 Months for Mild Stroke Patients who received either 0.6 mg/kg or 0.9 mg/kg rt-PA.

The overall distribution of mRS at 3 months showed no significant difference between the two groups (*p* > 0.05; Fig. 1). In addition, at 3 months, 73.9% of the patients in the 0.6 mg/kg dose group had an excellent functional outcome (mRS of 0 to 1); this was a slightly higher proportion than for patients in the standard dose group (71.0%) (*p* > 0.05) (Table 2). Our univariate analysis showed that only the baseline NIHSS score (*p* = 0.001), latency between onset and rt-PA (*p* = 0.001), and LDL-c (*p* = 0.004) were significantly different between subgroups with favorable and unfavorable functional outcomes, while the variables of gender (*p* = 0.885), age (*p* = 0.923), coronary artery disease (*p* = 0.909), hypertension (*p* = 0.772), diabetes (*p* = 0.797), atrial fibrillation (*p* = 0.463), smoking (*p* = 0.693), systolic blood pressure (*p* = 0.117) and glucose level (*p* = 0.342) were excluded from the multivariate logistic regression analysis. The multivariate logistic regression analysis used favorable functional outcome as the independent variable, and showed that dose (OR = 0.303; 95% CI 0.008–10.949; *p* = 0.514) was not associated with a favorable functional outcome after the model was adjusted for the following variables: baseline NIHSS score (OR = 0.482; 95% CI 0.299–0.776; *p* = 0.003), latency between onset and rt-PA (OR = 0.986; 95% CI 0.975–0.997; *p* = 0.009), and LDL-c (OR = 0.471; 95% CI 0.269–0.826; *p* = 0.009).

### In-hospital cost

As expected, the total cost during hospitalization for the 0.6 mg/kg dose group was significantly less than that for the standard dose group (3,401.7 USD vs 4,157.4USD, respectively; *p* < 0.01) (Table 2).

## Discussion

Our study showed that mild stroke patients who received a 0.6 mg/kg dose of rt-PA had equivalent favorable clinical outcomes and similar adverse complications to patients who received the standard 0.9 mg/kg dose but at a substantially reduced cost. To the best of our knowledge, this study is the first to evaluate a 0.6 mg/kg rt-PA treatment in a cohort of mild stroke patients, who have generally been excluded from rt-PA treatment.

Currently, there is no consensus on the use of rt-PA for certain mild stroke patients as studies have reported inconsistent results. Enormous efforts have been made to reach a definitive conclusion on the efficacy of thrombolysis for mild stroke, including an ongoing randomized trial with rt-PA^21^ and a study that evaluated various thrombolytic agents^22^. Here, we explored whether a lower dosage of rt-PA would be an alternative worth further studies for mild stroke patients.

We were previously concerned with the safety profile of a 0.6 mg/kg rt-PA treatment for patients with mild stroke. According to our findings, sICH was relatively infrequent in the 0.6 mg/kg dose group and even slightly less frequent than in the 0.9 mg/kg dose group and than in that reported elsewhere for a group with a higher NIHSS score who received a 0.9 mg/kg dose^23^. The reported difference in sICH incidence between the two groups in our study is supported by another study that randomized patients to either 0.9 mg/kg or 0.6 mg/kg rt-PA regardless of NIHSS score; the authors showed that the rate of sICH in the 0.9 mg/kg dose group was twice than that in the lower dose group^24^. Moreover, in our study, there were no reports of systemic hemorrhage during the course of thrombolysis in the 0.6 mg/kg dose group, while there were 3 cases of gum bleeding in the standard dose group (although none had severe consequences). However, both sICH cases in the 0.6 mg/kg dose group were fatal ICH cases that led to in-hospital death, while only 1 patient died among the three sICH cases in the 0.9 mg/kg dose group. Therefore, there was a slightly higher in-hospital mortality in the 0.6 mg/kg dose group. The three patients who died following the fatal bleeding were in their fifties, presented with hemiparesis, and denied having a previous history of hypertension but had extremely high systolic blood pressures (all BP >210 mmHg) at presentation. In addition, these three patients initially experienced significant recovery during the thrombolytic treatment before they dramatically deteriorated soon after thrombolysis. It is commonly accepted that age and the NIHSS score at baseline are among the most important risk factors for and predictors of sICH after thrombolysis treatment^25^. However, because the three fatal ICH cases did not fulfill these two conditions, we postulated that there were other underlying mechanisms.

It was worth mentioning that all three patients presented with steeply elevated BP at admission; based on the 2013 AHA/ASA guidelines^1^, the BP was brought down below 185/110 mmHg before the rt-PA treatment and was maintained below 180/105 mmHg with a continuous intravenous pump of sodium nitroprusside once the therapy was given. It is not uncommon for patients with acute ischemic stroke to present with a high BP: for example, there is a report of a mean systolic BP of 160.1 mmHg at admission^26^. It has also been well established that high blood pressure at presentation is associated with an elevated risk of sICH with intravenous alteplase^27^. Thus, it is possible that the excessively high admission BP in these three cases may largely account for the sICH. However, we were unable to uncover the fundamental mechanism underlying the relationship between an abnormally high BP and sICH because the patients’ relatives refused an autopsy.

Although there were two deaths in the 0.6 mg/kg dose group and so the mortality of this group seems higher than that of the 0.9 mg/kg dose group, this difference was not significant (*p* > 0.05). We cannot draw a definitive conclusion on the after-thrombolysis death based on the current information. We also cannot simply assume a relationship between the lower dosage of rt-PA and the higher incidence of fatal bleeding in the 0.6 mg/kg dose group as the common consensus is that the higher the dose of rt-PA, the higher the risk of sICH. Furthermore, as the patients with a higher sICH risk were more likely to receive a lower rt-PA dose in our study, this might be another explanation.

In conclusion, it seemed that the 0.6 mg/kg dose regimen was comparatively safe for the mild stroke patients with respect to the rate of sICH. The two fatal ICH cases in the 0.6 mg/kg dose group showed that the reduced dose of rt-PA would not lead to a dramatic decrease in fatal bleeding. And we are still challenged by the potential risk of severe outcomes following the administration of rt-PA. Those who present with extremely high blood pressure should be treated with special caution, and the optimal management of BP before and after the use of rt-PA for these patients may need further consideration.

Regarding clinical outcome, our results also demonstrated that a significant proportion of the patients who were treated with 0.6 mg/kg rt-PA reached a favorable functional outcome at 3 months. Overall, at 3 months, 73.9% patients who received the lower dose of rt-PA had an excellent functional outcome (mRS 0 to 1), which was a similar outcome to those who received a standard dose (71.0%). This result shows that the effectiveness of thrombolysis for mild stroke is not necessarily compromised by the lowered dosage of rt-PA as has previously been assumed; theoretically, a lower ‘clot volume’ might justify a lower dose requirement of rt-PA because of the small vessel occlusion commonly observed in mild stroke. We did not expect a more favorable outcome from the 0.6 mg/kg dosage as this would be counteracted by the ceiling effect. Notably, we did not include a group of mild stroke patients without rt-PA in the current study and were, therefore, unable to compare the outcome of such a group to the two treatment groups. Thus, we cannot draw a conclusion regarding whether the patients who received the 0.6 mg/kg dose regimen had a better functional outcome than those who received only conservative treatment.

END is a serious clinical event that is largely unpredictable and strongly associated with poor outcome^28^. Previous studies have demonstrated that the causes of most END cases after rt-PA could hardly be determined, with a few attributed to sICH, malignant edema, and early recurrent stroke, which were also confirmed in our study. Unlike previous studies, however, our patients suffered a higher incidence of END, i.e., almost 20% of patients from both groups, while only 7–10% has been reported in other studies^28^,^29^. Based on the possible mechanisms reported by previous researchers^28^,^29^, the higher END incidence in our study could be attributed to less use of antiplatelet agents before treatment, a lower NIHSS score at admission, and a longer latency between the onset and rt-PA treatment. Nevertheless, there was still no significant difference between the two groups in the incidence of END.

The last important finding of our study was the markedly lower cost for the in-hospital treatment of the 0.6 mg/kg dose group. In accordance with the standard strategy (0.9 mg/kg body weight, to a maximum of 90 mg), up to 70% of our patients should have consumed more than 1 vial of Actilyse^®^ (only 50 mg vials are available in mainland China); the standard strategy would cost twice as much as the 0.6 mg/kg regimen, as most of the patients receiving 0.6 mg/kg dose, if not all, required at most a single 50 mg vial of Actilyse^®^. The cost-saving strategy may be a more acceptable option for those partially covered by the healthcare system, those with low incomes or those living in less developed regions^30^. Additionally, these considerations could improve the risk-benefit profile.

There are several limitations of our study that should be noted. First, the retrospective, nonrandomized design of our study limits the generalizability of the outcomes to real-world circumstances. As can be observed, this study was confounded by the reasons for which patients were offered the lower dose, although the basic characteristics in each treatment group were well matched. Additionally, we were unable to accurately determine the mortality of lower rt-PA dose or uncover the relationship between the lower rt-PA dosage and sICH given the few cases of (fatal) sICH and our relatively small study population; thus, the related analyses should be interpreted with caution. Accordingly, randomized controlled clinical trials with large sample sizes are needed to detect possible differences between the different treatment strategies for the mild stroke population. Second, in our study, ‘mild stroke’ was defined as a NIHSS score ≤5 at admission in accordance with prior studies; however, there is no consensus on the cut-off for the NIHSS score to define ‘mild stroke’^31^, and it is possible that a different NIHSS score could suggest varied stroke mechanism and lead to a different reaction to the rt-PA.

## Conclusion

We believe that a low-dose rt-PA regimen for mild stroke patients could be an alternative worth studying in the future. The optimal dose for acute ischemic stroke still needs to be re-evaluated as this has not yet been fully studied. Additionally, the results from recently published trials for intra-arterial treatment of acute ischemic stroke drew a substantially positive conclusion^32^,^33^; a new treatment should also be taken into consideration if the ischemic lesions are caused by a causative occlusion of the internal carotid artery or proximal middle cerebral artery. Thus, further studies to determine a definitive strategy for mild stroke need to be conducted in the future.

## Additional Information

**How to cite this article**: Yang, J. *et al.* A Retrospective Study of thrombolysis with 0.6mg/kg Recombinant Tissue Plasminogen Activator (rt-PA) in Mild Stroke. *Sci. Rep.* **6**, 31344; doi: 10.1038/srep31344 (2016).

## Figures and Tables

**Table 1:** General Characteristics of the Study Population by Treatment Group.

	0.6 mg/kg (n = 46)	0.9 mg/kg (n = 62)	***P***
Age, years, median (IQR)	65.50 (59.00–70.00)	64.50 (59.00–72.25)	0.988
Gender, male, %	56.5	54.8	0.862
Personal history, %			
Hypertension	67.4	74.2	0.440
Diabetes mellitus	28.3	33.9	0.535
*Atrial fibrillation	10.9	12.9	0.748
Coronary artery disease	8.7	12.9	0.491
Data on admission			
Baseline NIHSS score, median (IQR)	4 (3–5)	3 (2–5)	0.237
Onset to rt-PA, min, mean (SD)	193.40 (56.09)	190.21 (50.89)	0.761
SBP, mmHg, mean (SD)	150.26 (20.20)	151.15 (21.83)	0.830
Blood glucose, mmol/L, median (IQR)	7.75 (6.52–9.31)	7.02 (6.01–8.42)	0.099
LDL cholesterol, mmol/L, mean (SD)	3.07 (0.85)	3.26 (1.01)	0.340
TOAST classification			0.930
Large artery atherosclerosis, %	26.1	25.8	
Cardioembolic stroke, %	8.7	8.1	
Lacunar infarction, %	34.8	35.5	
Undetermined stroke, %	28.3	27.4	
Other, %	2.2	3.2	

^*^Any diseases that could lead to cardioembolic stroke were included.

**Table 2:** Clinical Outcomes of the Two Treatment Groups.

Outcomes	0.6 mg/kg	0.9 mg/kg	***P***
END within 24 hours, %	19.6	17.7	0.809
sICH, %	4.3	4.8	0.905
In-hospital mortality, %	4.3	1.6	0.792
mRS at day 90, median (IQR)	1 (0–2)	0 (0–2)	0.121
mRS 0–1, %	73.9	71.0	0.735
*Total cost, median (IQR)	3,401.7 (3,115.9–3,684.5)	4,157.4 (4,024.6–4,400.7)	＜0.01

^*^Chinese Yuan were converted into USD according to the average exchange rate for 2013 to 2015 (1$US dollar ≈ 6.18 Yuan).
